# Increased Biocompatibility and Bioactivity after Energetic PVD Surface Treatments

**DOI:** 10.3390/ma2031341

**Published:** 2009-09-21

**Authors:** Stephan Mändl

**Affiliations:** Leibniz-Institut für Oberflächenmodifizierung, Permoserstr. 15, 04318 Leipzig, Germany; E-Mail: stephan.maendl@iom-leipzig.de; Tel.: +49-341-235-2944; Fax: +49-341-235-2313

**Keywords:** ion implantation, surface topography, biocompatibility, wear, corrosion

## Abstract

Ion implantation, a common technology in semiconductor processing, has been applied to biomaterials since the 1960s. Using energetic ion bombardment, a general term which includes conventional ion implantation plasma immersion ion implantation (PIII) and ion beam assisted thin film deposition, functionalization of surfaces is possible. By varying and adjusting the process parameters, several surface properties can be attuned simultaneously. Extensive research details improvements in the biocompatibility, mainly by reducing corrosion rates and increasing wear resistance after surface modification. Recently, enhanced bioactivity strongly correlated with the surface topography and less with the surface chemistry has been reported, with an increased roughness on the nanometer scale induced by self-organisation processes during ion bombardment leading to faster cellular adhesion processes.

## 1. Introduction

The similarity of patterns formed by lifeforms and the inorganic world, either subtle or complex, was first recognized by the Scottish zoologist D’Arcy Wentworth Thompson in 1917 [1]. For example, physical causes lead to the formation of honeycombs, similar to hexagonal, basalt rock formation or soap bubble arrays, mineral cages of the radiolarian *Aulonia hexagona* resembling buckministerfullerenes [2]. It is nowadays established that genetic, biochemical, chemical and physical forces are simultaneously active in living organisms. Thus, multiple pathways exist to activate biological processes, which are independently addressed in clinical applications, including, for example, drug eluting stents [3], nitric oxide releasing materials [4] or artificial biomaterial surfaces [5]. In this review, the focus will be on observed deficiencies and possible improvements for metallic biomaterials using purely physical surface modification, i.e., employing energetic PVD surface treatments.

The common history of metallurgy and artificial biomaterials dates back more than 3,000 years to the Bronze Age and before [6]. Romans, Chinese and Aztecs employed materials such as gold for dentistry [7,8], while the materials used by dentists in the Neolithic are still unknown [9]. Additionally, wooden teeth and glass eyes were utilized early on in this first generation of biomaterials. Nevertheless, poor understanding of materials science and biochemistry resulted in very low success rates. Around the middle of the last century, general use of materials originally developed for military applications, including novel metal alloys, ceramics and polymers, became widespread, even for biomaterials. Pioneering applications encompass total hip replacements consisting either from high density polyethylene and metal as developed by Charnley [10] or metal-metal-components by McKee and Watson-Farrar [11]. Nevertheless, biocompatibility had been a rather crude concept at that stage and high failure rates were present [12].

In the following decades, tremendous advances in biochemistry and materials science, including surface science occurred, allowing a description of processes and mechanisms on an atomic scale, inside living organisms as well as inside materials, and thus an understanding of the interaction between artificial implants and the host tissue [13,14]. Consequently, an integrated approach by physicists, materials scientists, biologists and physicians throughout the 1980s led to the development of about 50 implantable devices using more than 40 different materials, including shape-memory alloys [15,16]. This first generation of biomaterials was characterized by suitable physical properties showing a minimal toxic response.

A major shift in emphasis towards bioactivity resulted in the advent of tissue engineering in the early 1990s. A combination of cells, biologically active molecules and carrier material should elicit a controlled action and reaction in the physiological environment. Hence, second-generation biomaterials were designed to replace biological functions and to be resorbable or bioactive [17,18]. After the turn of the century regenerative medicine was formed as stem cell engineering converged with tissue engineering, shifting the focus even further towards human cells and away from artificial biomaterials [19]. This third-generation of biomaterials distinguishes between external creation of tissue and materials that evoke specific cellular healing responses [20,21].

**Figure 1:**
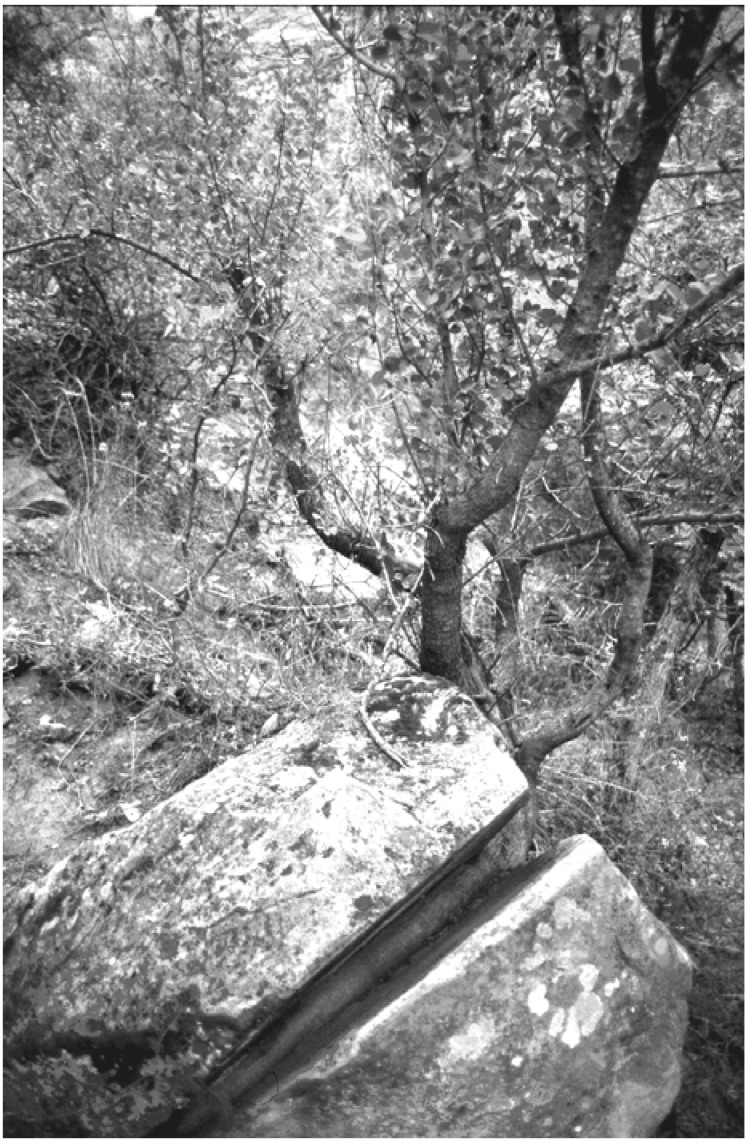
Example of living tissue interacting with non-adaptive materials: The soft tree visible in the picture was able to cut deep into the hard rock.

The interdisciplinary task of improving the biocompatibility is complicated by the boundary conditions imposed by the living tissue. All biomaterials are directly employed at the interface between organic and inorganic functions. Organisms employ less than 10 elements from the periodic table with additional 10–20 trace elements. The defining characteristics are hierarchical structures across several orders of magnitude down to single molecules, multifunctionality and self-healing capabilities [22,23,24]. Conventional materials engineering uses a top-down approach, in contrast to the bottom-up organization in living tissue. Adaptation to environmental influences (cf. Figure 1) and self-repair capabilities are other advanced capabilities of natural materials.

However, tissue engineering is still a young technology with limitations, especially for load bearing applications. Thus, artificial biomaterials, which are the only option for some application, still encompass a large sector in the medical devices sector. However, despite continuous improvements, revision rates of up to 5–15%, depending on the application, are nowadays still present [25]. Several failure causes related to the materials surface can be loosely grouped into four different categories: (i) corrosive failure; (ii) abrasive wear; (iii) adverse topography; (iv) non-hydrophilic surface.

Presently, metallic biomaterials define a large worldwide market—still with double-digit growth expected for at least five years—with joint reconstruction and trauma fixation as major constituents with a combined volume of more than $15 billion in 2008 [26,27]. Here, a replacement by organic material grown by tissue engineering is not achievable on a medium timescale. Presently, different strategies are employed to address the mentioned failures causes separately [5,28].

Hydroxyapatite coatings are often used as porous coatings to facilitate bone ingrowth [29]. Alternatively, blasting can be used to achieve a certain surface roughness. Polymer coatings are often employed used for articulating surfaces or for blood contact as they have a low friction coefficient and are non-thrombogenic [30]. Additionally, antibacterial activity of metallic implants has been achieved without reverting to biochemistry, either by incorporating active Ag nanoparticles into the surface region [31] or using photoactive surfaces [32,33]. Chemical etching is another emerging alternative to create nanostructured surfaces with increased biocompatibility [34,35]. Furthermore, bioresorbable alloys, especially magnesium-based systems, are gaining importance and will see clinical use when the dissolution rate is eventually decreased [36].

Nevertheless, an urgent requirement exists at present to improve the biocompatibility of existing implants at a reasonable cost level. Thus, instead of several subsequent surface modifications addressing independently the mentioned problem groups, a single process, addressing all possible causes leading to failure at once and simultaneously is desirable. Using surface treatments with energetic ions, firmly established in research and production environments, is one possibility to modify the interaction between biological tissue and biomaterial. Thus, a specifically tailored surface can be provided with synergistic effects encompassing several interaction mechanisms simultaneously. Presently, investigations directed towards such synergies are rare, however their frequency is increasing. At the same time, research focusing on, e.g., improved wear resistance, often inadvertently modifies the corrosion resistance as the surface chemistry is changed without presenting corresponding experimental results.

Energetic surface modification with ions can be one possibility, as a multitude of surface properties, including topography, chemistry and tribology are addressed simultaneously. Furthermore the surface topography is modified in a “bottom-up” approach without any templates as self-organization processes are activated by the impinging ions. At the same time, a large parameter space is available by varying ion species, energy and fluence, which is benefit and bane simultaneously. A detailed surface modification according to specific biological requirement is possible. At the same time, the process reproducibility, transferability and scalability has to be scrutinized closely.

In this review, the underlying principles of energetic surface treatments are discussed in Section 2, concentrating on coating and implantation processes and subsequent changes in the topography. Section 3 will give a short introduction into biochemical surface interactions and how modified biomaterial surfaces can change them. The main part, Section 4, is dedicated to a presentation of the state-of-the-art of surface modification of metals with energetic ions, concentrating on stainless steel, CoCr alloys, titanium alloys, NiTi shape memory alloys and bioresorbable magnesium alloys.

## 2. Energetic Surface Treatments

### 2.1. Ion Implantation & Physical Vapor Deposition

Energetic surface modifications can be roughly categorized using the incident ion energy as parameter. At low kinetic energies below approximately 10 eV, depending on the ion-target combination and the angle of incidence, the incoming ion (or neutral atom, respective molecule, as the charge state is not important in this consideration) will be stopped within the first monolayer or even at the surface itself if the energy is too low to create atomic displacements inside the target material [37]. Thus, a deposition onto the surface can be observed.

**Figure 2:**
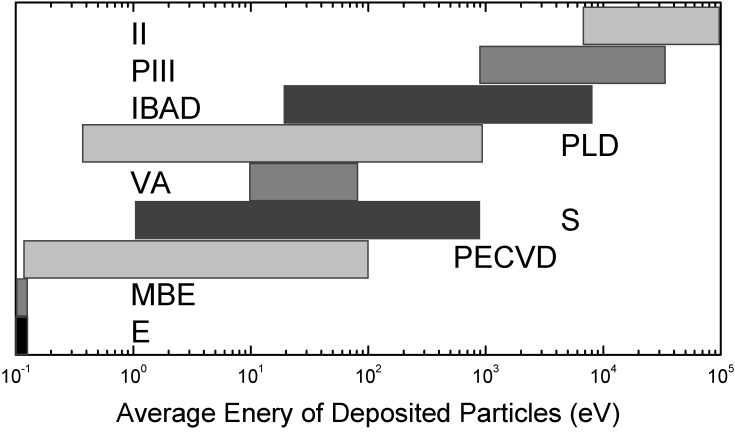
Spread of average energy per incoming particle for different processes: evaporation (E), molecular beam epitaxy (MBE), plasma enhanced chemical vapor deposition (PECVD), sputter processes (S), vacuum arc (VA), pulsed laser deposition (PLD), ion beam assisted deposition (IBAD), plasma immersion ion implantation (PIII) and conventional ion implantation (II). An energy of 1 eV corresponds to a temperature of about 11,600 K.

**Figure 3:**
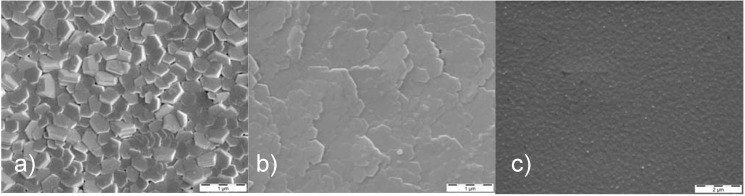
Surface morphology of Mg films deposited at room temperature with a rate of about 1 µm/h by (**a**) magnetron sputtering, average energy ~8 eV, (**b**) ion beam sputtering, average energy ~12 eV, and (**c**) vacuum arc deposition, average energy ~25 eV [44].

Typical physical vapor deposition (PVD) processes encompass, with increasing energy per deposited particle: evaporation, reactive sputtering, vacuum arc deposition and ion beam assisted deposition (IBAD) techniques [38,39,40]. The systematic variation of the ion energy from thermal energies near 0.05 eV up to 500 eV or more in these processes is depicted in Figure 2, with typical surface morphologies depicted in Figure 3. Correspondingly, the films become denser with a reduced void fraction, caused by an enhanced surface mobility [41] and larger crystallites due to Ostwald-ripening of the formed nanocrystals [42]. Large efforts have been made to use the resulting thin films for biomechanical coatings [43]. At the same time, a higher average energy per deposited particle leads to a reduced surface roughness as the enhanced surface mobility leads to a mass transport on the surface.

A related method to produce coatings with electrical discharge events is called micro-arc oxidation (MAO) or plasma electrolytic oxidation (PEO) [45,46], where localized micro-arc discharges are formed on the surface of samples inside electrolytic solutions at voltages of 200 V and beyond. Thus, thick, porous and micro-structured are formed oxides incorporating additional elements from the electrolyte [47,48]. The voltage used during this process is much higher than in conventional anodic oxidation, which results in rather flat surfaces.

For higher ion energies, ion implantation, developed in the early 1950s by Shockley in the Bell Laboratories [49,50] and nowadays indispensable in the semiconductor industry, leads to a controlled insertion of foreign atoms below the surface. The range of the ions, and hence the modification depth, can be systematically changed from a few nanometers to several micrometers by varying the ion energy from 10 keV to more than 1 MeV. In general, a solid solution of the inserted atoms in the host matrix is present at low implanted doses. With increasing dose, a supersaturation followed by the nucleation and growth of precipitates, which finally coalesce into closed layers, is occurring. In addition to the possibility of implanting any chemical element into any matrix, ion implantation is characterized by the possible formation of metastable phases far from the thermodynamic equilibrium [51,52].

This insertion of energetic ions is always correlated with the deposition of their kinetic energy in the surface region. On the one hand, this energy leads to a local and temporal increase of the mobility (on a timescale of picoseconds and volumes comprising around 100 atoms) so that phase formation processes are not restricted by equilibrium thermodynamic. However, rapid cooling occurs with cooling rates of more than 10^12^ K/s within these ion tracks. At the same time, a majority of this energy is transferred into the formation of defects, interstitials and vacancies, which leads to displacement of atoms, intermixing and sputtering of particles from the surface [53]. Besides application in the semiconductor industry [54], ion implantation into metals was employed early for tribological applications including biomaterials, e.g. artificial hip implants [55].

In conventional ion implantation, the ions are produced in a plasma source, extracted, separated according their mass, accelerated and finally implanted into the sample. This extraction and acceleration result in an ion beam with a small diameter, so that a beam steering and target manipulation system are necessary for large and complex shaped samples, increasing the treatment time and the treatment costs. Using broad beam ion sources without mass separation, simultaneous implantation into large flat surfaces is possible [56].

By inserting the target directly into the plasma source, an implantation into arbitrarily shaped objects is possible. This process, called plasma immersion ion implantation (PIII), was developed independently in the US and Australia about 20 years ago [57,58,59], is nowadays a versatile method for surface modification, where negative high voltage pulses are applied to a substrate which is immersed in a plasma. Thus, the positive ions are extracted from the plasma and accelerated towards the whole surface simultaneously. This method combines the advantages of plasma treatments, like PVD processes or plasma nitriding [60], and ion implantation in one method: the modification of complex shaped surfaces without beam steering and target manipulation with high energy ions for the formation of new or metastable phases is possible in a short time and cost-effective way.

In metal plasma immersion ion implantation and deposition (MePIIID) [61], a combination of vacuum arc deposition and PIII, a cathodic arc is used as a source for positive metallic ions resulting in a supersonic plasma stream emanating from the cathode with an ionization ratio of nearly 100% and an average charge state of 2–3 [39,62]. On the upstream side of the sample no presheath is present, while the plasma density drops by a factor of 5–10 on the downstream side, depending on the applied voltage. Another effect of the strongly directed stream is a very strong decrease of the layer thickness on areas not exposed to the plasma stream [63]. An additional problem, which has to be considered, is the generation of macroparticles, especially for metals with a low melting point or carbon [64], which have to be eliminated either by a filter [64] or by a shield in front of the arc [65].

For both deposition and implantation processes, the kinetic particle energy is ultimately converted into heat, which must be removed either by radiative or convective cooling from the substrate. Higher particle energies and higher deposition rates lead to stronger increases in the temperature. At a deposition rate of 1 nm/s, corresponding to a particle flux of about 6 × 10^15^ cm^-2^s^-1^ and an average energy of 1 keV, about 1 W/cm^2^ is generated. Higher particle energies require respective compromises in deposition rates and vice versa.

### 2.2. Evolution of Surface Topography under Energetic Particle Bombardment

Surface bombardment with energetic particles leads to surface erosion, a phenomenon named sputtering, which was first discovered on cathodes of electric gas discharges [66]. In a qualitative description of the sputtering processes for keV ions, atomic collision cascades are initiated in the surface region [67]. Upon receiving a momentum component towards the surface and an energy which is larger than the surface binding energy, a target atom can be sputtered.

**Figure 4:**
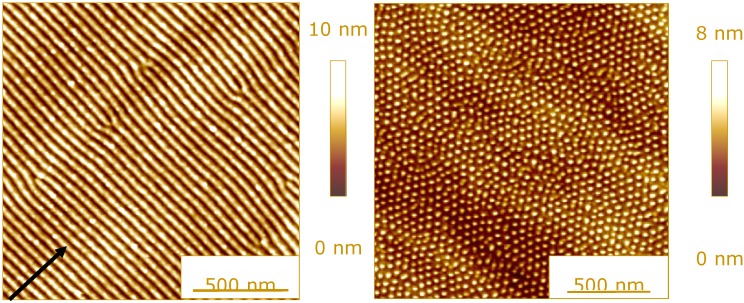
Surface topography after ion bombardment: (**a**) Si surface, 1200 eV Ar^+^, 15° off-normal; the arrow indicates the projected ion beam direction. (**b**) Ge surface, 2000 eV Xe^+^, 20° off-normal [72].

Sputter thresholds exist for primary particle energy with values of about 20–40 eV, depending on the combination of projectile and target. Beyond the threshold a fast increase of the sputter yield is observed until a maximum at 5–50 keV and a slower decline for even higher ion energies. Sputter yields may be calculated using equations developed by either Yamamura [68] or Eckstein [69]. In addition to this physical sputtering, enhanced or reduced sputtering may be instigated by chemical binding forces, i.e., chemical sputtering [70,71].

This removal of surface atoms by sputtering, together with radiation damage and the insertion of the foreign atom into the host material leads in general to a modification of the surface topography. For semiconductors, nanostructures including regular ripples [73,74] or dot arrays [75,76] can be observed, in addition to atomically smooth surfaces [77] or disordered nanostructures [78], as shown in Figure 4 [79]. These former structures are explained by a competition of sputtering and surface relaxation mechanisms [80]. A similar behavior can be found for insulators and metals.

**Figure 5:**
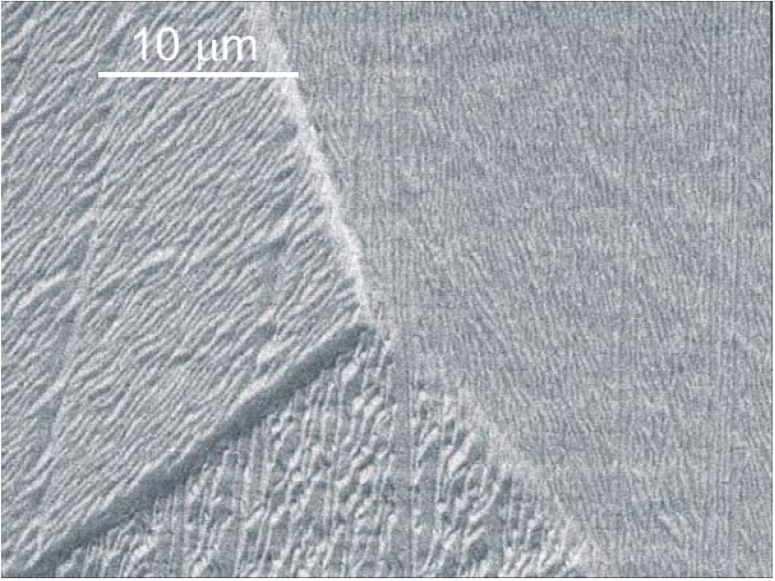
SEM micrograph of Al surface after sputtering with 5 kV Cs^+^ for a dose of 6 × 10^17^ at./cm^2^ at an incident angle of 60° [83]. A triple grain boundary with different absolute sputter yields and different surface roughness for each grain is visible.

**Figure 6:**
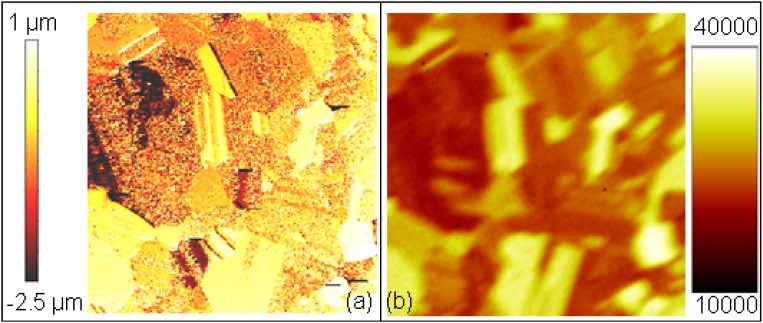
(**a**) Surface topography image. (**b**) secondary ion image (intensity corresponds to counts/pixel). The image size is 300 × 300 µm^2^. The microstructure determines local sputter rates and secondary ion yields, however no direct correlation between them is possible.

For polycrystalline materials, a non-uniform erosion is observed, with differently oriented crystallite eroded at different rates and higher erosion observed for grain boundaries [81,82], with strong dependencies on target material, ion species and ion energy. Typical examples for such effects are shown in Figure 5 and Figure 6 for Al [83] and CoCr alloy which were subjected to an ion beam during SIMS depth profiling. In the former case, steep edges are observed at the grain boundaries, together with a strongly varying nano-roughness on differently oriented crystals. The latter example highlights the expected correlation between sputter removal and secondary ion count rate. Additionally, differences in sputter yield caused by twin boundaries can be observed. In total, a linear increase of the root mean square roughness *R_q_* with ion fluence is observed initially, whereas a slower increase but no saturation is observed beyond 10^18^ cm^-2^.

At elevated temperatures, further diffusion and phase formation processes, in addition to surface mobility of atoms, may be activated, which will result in further changes in the surface topography. Important systems here encompass transition metals modified with carbon, nitrogen or oxygen [84], where ion implantation, providing mobile atoms within the material, high supplements conventional thermal nitriding or oxidizing processes. Thus, lower process temperatures can prevail for ion implantation than for gas or plasma thermochemical treatments, where dissociation and transport through the surface barrier are advanced by elevated temperatures in the range from 600 to 1,200 °C [85]. At the same time, hard and wear resistant metal carbides, nitrides and oxides provide excellent surface protection layers [86,87].

**Figure 7:**
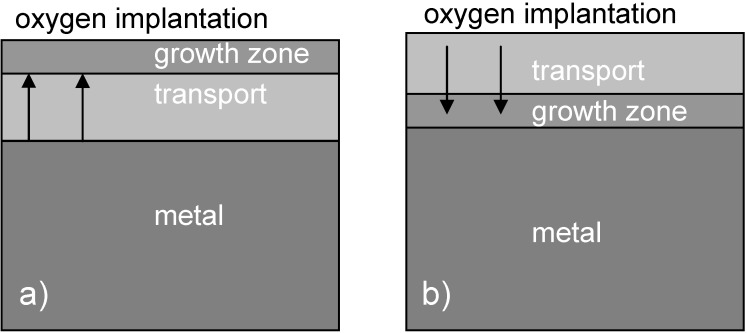
Schematic presentation of layer growth processes: (**a**) Faster transport for metal cations, growth near the surface. (**b**) Faster transport for oxygen anions, layer growth at the interface.

Depending on the ion fluence, different types of diffusion processes will occur. At low fluences before secondary phases are formed, the implanted atoms diffuse inside the host matrix, while at higher fluences, additional compounds with a stoichiometric composition are formed, with either dominating anion or cation mobility—or diffusion of both species [88,89]. For closed surface layers, the growth of these layers occurs via diffusion limited transport across the layer with the formation enthalpy acting as driving force. As a result, an inverse parabolic increase of the layer thickness *d* is observed with time *t*, i.e., *d* ∝ *t*^1/2^. Depending on the species with the higher mobility, the growth process is concentrated on the surface or the interface (see Figure 7). It has to be pointed out that the ion range is normally much smaller than the respective oxide, nitride or carbide layer.

As polycrystalline materials are commonly used in technical applications, grain boundary diffusion processes have to be considered in addition to bulk diffusion processes [90,91]. While, enhanced atomic mobility in grain boundaries was recognized already in the 1920s, quantitative studies were started only in the 1950s, beginning with Fisher’s model [92], which distinguishes between a diffusivity for the grain boundary region and a diffusivity for the rest of the crystal. For self-diffusion in metals, an Arrhenius-type temperature dependence is observed with similar pre-exponential factors but about only 50% of the activation energy for grain boundary diffusion compared to bulk diffusion [93].

**Figure 8:**
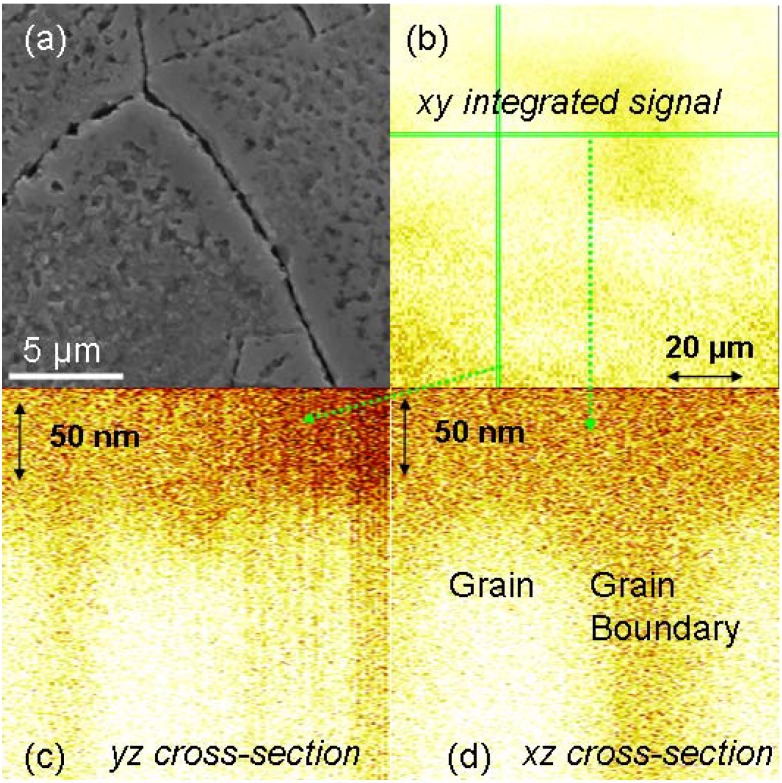
Grain boundary diffusion in Ni after oxygen implantation: (**a**) SEM viewgraph of the surface. (**b**) SIMS surface image (Ni ion signal). (**c**) SIMS cross-section along vertical line in (b). (**d**) SIMS cross-section along horizontal line in (b).

For compounds, the situation is much more unclear as point defects and dislocations in the bulk may enhance diffusion processes, while impurity segregation and phase precipitation at grain boundaries may decelerate diffusion processes. At the same time, different diffusion mechanisms inside grains and at grain boundaries cannot be excluded. A typical, well-understood example is Ni + O, where the dominant process is vacancy diffusion of Ni at grain boundaries, whereas oxygen grain boundary diffusion is even slower than Ni bulk diffusion [94]. Corresponding SEM and SIMS images obtained for polycrystalline Ni implanted with oxygen are shown in Figure 8, where the surface and the grain boundaries are clearly visible with a reduced signal intensity in SIMS.

**Figure 9:**
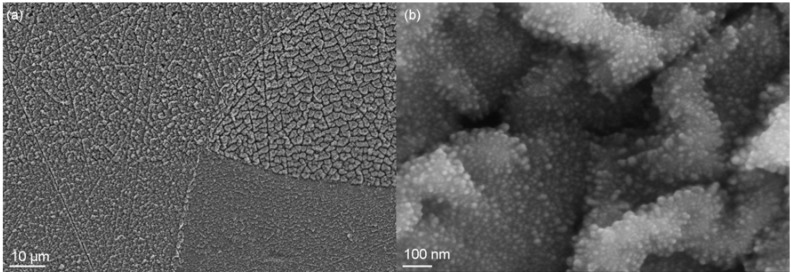
Surface topography of Al after nitrogen implantation at 350 °C: (**a**) SEM viewgraph at low magnification showing the influence of the original grain structure. (**b**) High magnification image detailing the diffusion and nucleation at grain boundaries.

Concentrating on systems with dominant outward transport of metal cations and subsequent nucleation and phase formation processes, pronounced nano-structured surfaces can be formed by ion implantation into metals at elevated temperatures. In the system Al-N, a pronounced outward diffusion of Al cations along grain boundaries is observed for nitrogen implantation into aluminum at moderate temperatures between 250 and 400 °C [95]. At the same time, variations in the orientation dependent sputter yield lead to strong topography and roughness dissimilarities between single grains, as shown in Figure 9(a) [83]. As the melting point of AlN is much higher than that of Al with a corresponding lower mobility inside AlN grains, outward growth of the resulting AlN surface layer is occurring predominantly along the grain boundaries of the AlN micro and nano-crystals, leading to a highly corrugated surface structure where it cannot be ascertained that a closed nitride layer is present (cf. Figure 9(b)). At the same time, this surface incidentally exhibits strong antireflection properties. Furthermore, the fast outward diffusion of Al leads to the formation of voids below the modified surface layer, thus compromising the adhesion [96].

**Figure 10:**
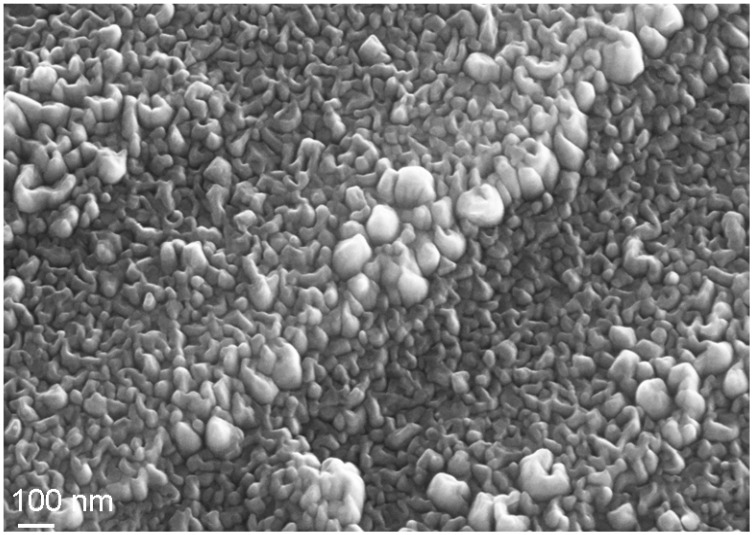
SEM viewgraph of grinded Ti after oxygen PIII at 450 °C for 30 minutes.

**Figure 11:**
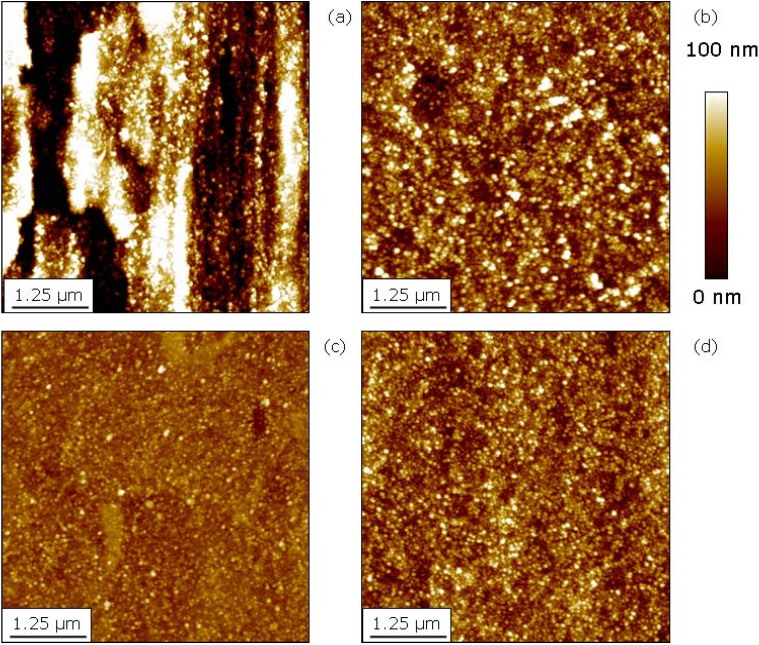
Surface topography measured with AFM after oxygen PIII at 450 °C for 30 minutes: (**a**) grinded Ti. (**b**) grinded + polished Ti. (**c**) Ti6Al4V (polished). (**d**) NiTi (polished).

For the biomedically important class of Ti alloys, an outward diffusion of Ti is observed after both oxygen and nitrogen implantation, however with only moderate diffusion rates in the technologically important temperature range up to 500 °C [97,98]. At the same time, closed oxide and nitride layers are formed, respectively. Oxygen implantation leads to a significant increase in the surface roughness as the topography is dominated by small oxide crystals, consisting of TiO_2_ in the rutile phase as additional alloying elements as Ni in NiTi and Al or V are characterized by a much smaller formation enthalpy and are thus prone to segregation effects towards the bulk, depleting them in the oxide. The average crystallite size itself varies between some 20 nm near 250 °C (see Figure 10) and 80 nm near 500 °C [99]. At identical process conditions, no differences are seen for pure Ti (grade 2), Ti6Al4V (grade 5) [100] and NiTi [101], as depicted in Figure 11. Even material, which was only grinded before oxygen implantation shows the same topography on a nanoscale superimposed on the microstructure as fine polished base material.

In contrast, inward diffusion of nitrogen is observed for stainless steel and CoCr alloys with no significant enhancement at grain boundaries. Thus, the surface topography is only influenced by the original microstructure and the subsequent ion bombardment. No appreciable diffusion is observed for these two alloy classes after oxygen implantation below 500 °C, which is correlated with the corrosion and oxidation resistance of the alloys [102].

### 2.3. Comparison of Coating and Implantation Processes

**Figure 12:**
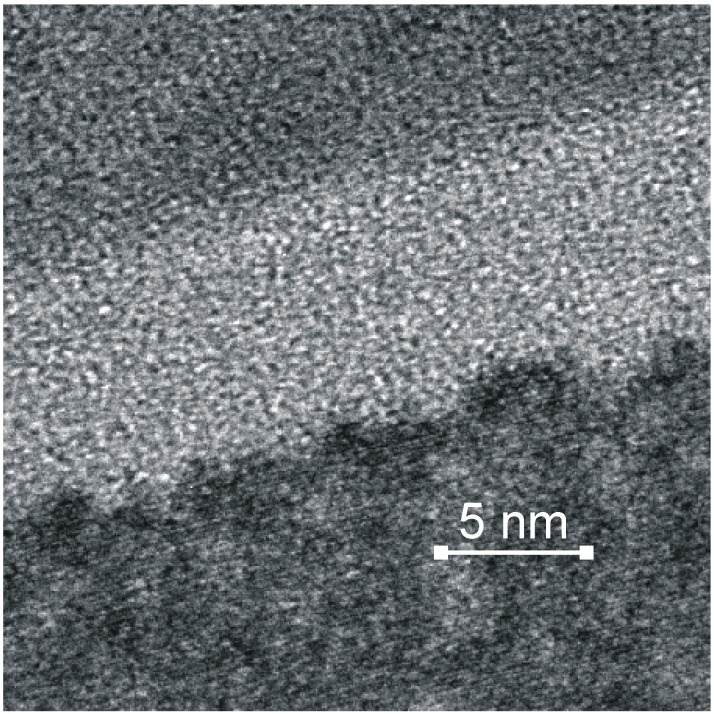
High resolution TEM cross section of the interface for a TiO_2_ thin film deposited on Si using additional ion bombardment during deposition (top TiO_2_, bottom Si) [107].

A large variety of coatings is produced commercially and in research laboratories [87], with thick layers of up to 1 mm presenting no technological problem. There is nearly no limit in the chemical composition and the tribological properties as a large knowledge reservoir was build during the last 50 years. Nevertheless, two problem classes still exist: adhesion and porosity. The interface between the substrate and the coating yet presents a weak point for two reasons. First, the atomic bonding between the coating and the base material depends critically on the precleaning process and the deposition temperature, with temperature beyond 250 °C necessary to maintain a good adhesion [103]. Furthermore, hard and brittle ceramic coatings may be applied on ductile and soft metals, thus leading to a more rapid delamination than for pairs with more similar elastic properties [104]. Secondly, corrosive attacks of the base material are facilitated by voids and pores always present in coatings [105], which is more critical for biomedical applications than in some other areas [106]. Increased ion energies during the deposition of coatings, or ion implantation, leads to atomically mixed interfaces (see Figure 12), even for 1 kV acceleration voltage and duty cycle of only 9% [107], whereas the reduction of porosity or grain boundaries necessitates higher energy deposition.

**Figure 13:**
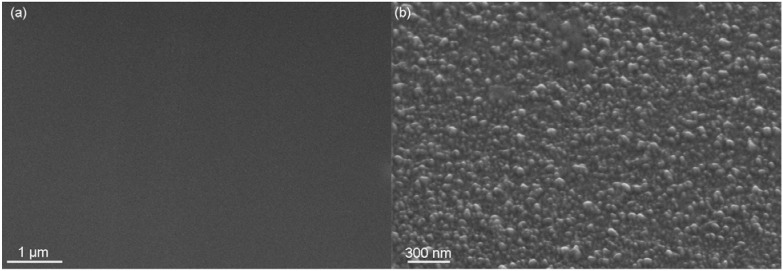
SEM viewgraph of TiO_2_ surfaces: (**a**) PVD deposition of TiO_2_ at room temperature. (**b**) PVD deposition of Ti at room temperature followed by oxygen ion implantation at 450 °C.

However, using ion implantation for surface modification is more complex as there is a restriction to the very few selected elements for which commercial ion sources (broadbeam or PIII) with attractive ion currents are available. At the same time, complex sputtering and diffusion processes limit the availability of suitable systems even more. One alternative is the evolution of a hybrid process with an initial ion assisted coating process for improved adhesion, a dedicated coating (which may consist of several single layers, multilayers or gradient layer) and, finally, a suitable surface topography formed by ion implantation processes. In Figure 13, the surface topography [108] of a TiO_2_ thin film produced by vacuum arc deposition (on the left) is compared with a TiO_2_ formed by deposition of Ti and subsequent oxygen ion implantation. But how do such surfaces topographies influence the biocompatibility and bioactivity? This will be the topic of the next section.

## 3. Biochemical Surface Interaction

The interaction between inorganic biomaterials and living cells is governed on the biomolecular side by the recognition of environmental cues which in turn affect cellular phenotype and function [109,110]. The corresponding physical and chemical surface properties can be categorized into four distinct groups with different interaction modes: (i) surface topography on the µm- and nm-scale, determining the adhesion of cells and receptor molecules [111,112]; (ii) the surface energy, respective electronic density of state at the surface, responsible for electron transfer and the distribution of the electrical potential, both parameters which may interrupt normal cell behavior [113]; (iii) the out-diffusion of metallic cations leading to toxic effects and apoptosis in the surrounding tissue, especially critical for Ni-containing metals [114,115]; (iv) the generation of wear particles in the micrometer and nanometer range and their transport [116,117]. However, a clear separation of the different mechanisms is often not possible as control experiments changing only one parameter without unintentionally varying another are rather difficult and synergy effects from combining two or more interaction paths cannot be excluded.

### 3.1. Topography

**Figure 14:**
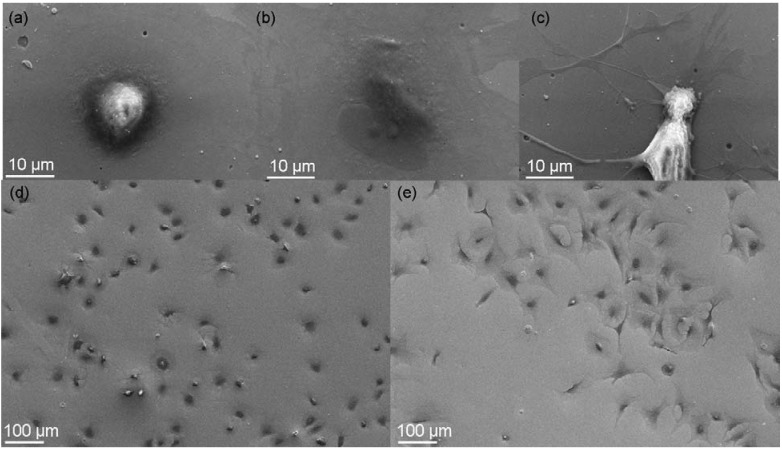
SEM images of osteoblast colonisation of a completely flat transition metal oxide surface, produced by a PVD process on a silicon wafer. (**a-c**) Typical transition stages of attachment and differentiation for individual cells. (**d**) surface after 1 hour. (**e**) surface after 4 hours.

The microenvironment of cells consists of the surrounding extracellular matrix (ECM), growth factors, and cytokines as well as neighboring cells. Cell surface receptors promote cell adhesion to the ECM scaffolding, with integrins as the major receptor class [118]. Hence, materials derived from natural ECM, such as collagen, providing natural ligands which promote cell attachment have an advantage as biomaterials [119]. However, additional strong effects of the surface topography on the micrometer and nanometer scale were observed, which resulted in enhanced protein and collagen production [120,121]. Albeit, supplementary influences of surface chemistry cannot be completely excluded as normal cell attachment and differentiation of osteoblasts can be also detected on flat surfaces as shown in Figure 14. Nevertheless, a delayed or inhibited reaction compared to a rough surface consisting of the same material was observed in this experiment.

When comparing different experiments on the influence of surface topography on the bioactivity, assiduous comparison of experimental details is necessary. Average grain size, defined by the distance between grain boundaries, and surface roughness, i.e., variations in topographical height, are normally not correlated. Additionally, different roughness values may be obtained using different measurement methods. Furthermore, the roughness itself depends on the instrumentation, especially the distance between single measurement points. A more informative value than the roughness is the power spectral density (PSD), which is calculated from the Fourier transform of 1D or 2D surface profiles. Beside the amplitude of surface fluctuations, the corresponding lateral wavelengths and orientation anisotropies can be inferred [122,123].

Recently, a basic understanding of the mechanisms responsible for this modulation of cell reaction was obtained. When grown on substrates with micron scale roughness, especially titanium substrates, an increased integrin expression of cells is observed [124], together with increased PKC signaling [125]. An active role of TGF-β1 by stimulating additional factors acting downstream is also reported in the literature [126,127]. Correspondingly, a higher degree of differentiation is observed for osteoblasts, including elevated levels of osteocalcin [128] and prostaglandins [129]. In additional experiment, stimulation of vasculogenesis [130] and decreased osteoclastic activity [131] was found.

For linear structure, the orientation of cells depends on the scale length of the structures and the cell type. For trenches or ridges with periods between 200 nm and 20 µm, the majority of focal contact points is on top of them with epithelial cells and sarcoma osteogenic cells oriented parallel to these ridges [132,133]. Similar results were observed for human hepatoblastoma cells on nano-grooved substrates with widths between 100 and 500 nm [134]. In contrast, for lamellae structures with a wavelength of 400–800 nm, an orientation of osteoblast filopodia perpendicular to the structures was observed [135]. Similarly, it is reported that different aspect ratios, i.e., height-to-width ratios, are favored by different cell types. Fibroblasts prefer deeper grooves for orientation than endothelial cells with the latter ones bridging grooves deeper than 0.35 µm on ground titanium surfaces [136]. At the same time, fibroblasts attach with their whole body, leading to better adherence than for endothelial cells that only show contact guidance the cytoplasm-poor margin. Furthermore, the inflammatory response of macrophages is modulated by a surface topography on the nanoscale [137].

Besides the topography, the surface texture can also influence the tissue response, presumably via the electronic surface structure or work function depending on the surface orientation. Higher cell attachment and proliferation of osteoblasts on Ti6Al4V were observed for surfaces oriented preferentially along (100) than for (110) oriented surfaces [138]. The former samples were obtained from rod material while the latter were cut from sheets. Thus the texture induced by different rolling processes or further mechanical deformations can result in pronounced changes in the biocompatibility.

Expanding the concept of surface topography from a two-dimensional surface to a three-dimensional surface leads to interconnected porous materials with a desired pore diameter between 200 and 1,000 µm [139,140]. Using a dilute HCl treatment on plasma sprayed porous titanium reduces the bone induction time from 12 months to about three months, comparable to porous CaP-based biomaterials [141]. Besides topographic etching effects, an additional chemical surface modification by Na removal and titania formation is postulated as cause.

### 3.2. Surface Hydrophilicity

In addition to the surface topography, the surface energy plays an important role in defining the bioactivity. Hydrophilic surfaces, in contrast to hydrophobic surfaces allow a better attachment and proliferation of cells [142,143,144]. The effect of conformational changes of adhesive proteins on altered biological properties is a well established fact [145,146]. Correspondingly, bioinert materials are preferred for blood contact, as the blood coagulation is directly correlated with fibrinogen adsorption [147,148], or surgical instruments [149]. Accordingly, such materials are suboptimal for bone implants. Acetabular sockets made from monolithic alumina show high failure rates due to loosening [150], which, in turn, can be alleviated by hydroxylation of the surface [151]. However, in addition to protein interaction with surfaces via conformational modifications, an interaction through integrin cell surface receptors is observed, again with hydrophobic surfaces showing a detrimental effect on the bioactivity [152,153].

### 3.3. Corrosion

Electrochemical corrosion, respective release of metal ions, is one of the major causes for unexpected bad biocompatibility of some implants, together with mechanical wear processes releasing particles [25,154,155]. Even scratches on the stem of hip implants created during insertion may lead to medical complications [156]. Even fast repassivation of surfaces, found especially for Ti alloys, and thick native oxide layers are sometimes not sufficient to reduce the leaching of these metallic ions to a satisfactory low level [157]. Depending on the alloy composition, different metals may be released from biomaterials.

Implants fabricated from Co-based alloys have been reported to produce elevated Co, Cr and Ni concentrations in body fluids [158,159]. There is concern of the cytotoxicity of V in Ti6Al4V, leading to many studies on metal release from Ti6Al4V and surface treatments to reduce the quantity of vanadium release [160,161]. Nickel is known as an allergenic and carcinogenic material, thus Ni containing metals are under special observation. Depending on the atomic binding, different Ni release rates are observed, with NiTi shape memory alloys showing lower release rates than Ni-containing stainless steel [16]. However, the long-term biocompatibility is still highly contentious with an increased risk of Ni^2+^ release [162]. In addition to the chemical composition, a strong influence of the environment, especially pH value and the fluid composition, on the corrosion mechanisms and metal release rates was observed [163,164]. Iron release from 316L stainless steel decreased with increasing pH, similar to the release of Ti from Ti alloys with very low amounts found for pH 4 and higher.

The toxicity of metals in physiological solutions has been widely investigated [165] and, correspondingly, the influenced biochemical pathways have been partially identified. Some of these elements are highly toxic (e.g., Co, Cu, Ni, V) with concentrations of less than 10 µM interfering with the normal cell differentiation and signaling pathways [166,167]. In general, Al^3+^, Co^2+^, Cr^3+^, Ni^2+^, Ti^4+^ and V^3+^ are cytotoxic at some concentration. At lower concentrations, Ni^2+^, Co^2+^, Ti^4+^, and V^3+^ affect the ROS cell metabolism, whereas Cr^3+^ and Al^3+^ do not show inhibitory effects [168,169]. Exposure of endothelial cells to CoCl_2_ leads to induction of cell death attributed to impaired integrin signaling, which depends on divalent metal ions—Ca^2+^, Mg^2+^, Mn^2+^ ‑ with their ion binding sites acting as target for the heavy metal ions [170]. However, at low Co^2+^ concentrations, increased vascularisation mediated by the HIF-1α (hypoxia-inducible factor) pathway normally activated by oxygen deficiency is observed in human endothelial cells [171]. Similarly, NiCl_2_ is able to induce an up-regulation of surface antigen expression in macrophages at low concentrations while impairing essential functions at higher concentrations [172]. Increased levels of Mg^2+^ do actually result in enhanced osteoblast function and proliferation [173].

In addition to local effects, long-range transport through either the blood stream or the lymphatic system can occur, leading to a noticeable concentration increase and storage of heavy metal ions in organs, especially liver, kidney and spleen [174,175]. Elimination in urine may occur, but this will remove only a fraction of the metals disseminated in the body from implants.

### 3.4. Wear

In addition to metallic ions, atomic clusters or particles generated by wear processes are known to cause adverse effects. Wear debris from implants are known to play a central role in osteolysis [176,177]. Stimulation of macrophages, fibroblasts, foreign body giant cells and T lymphocytes leads to the production of proinflammatory cytokines and other factors [178,179], which in turn induce RANK and RANKL expression by osteoblasts and marrow stromal cells [180,181]. While metal-on-metal hip joints show generally lower volumetric wear than metal-polyethylene or ceramic-polyethylene couples, up to 10^14^ particles with an average size below 1 µm are still released each year [182]. Aseptic loosening is not only reported for metallic particles but also for polymers, especially polyethylene [183].

## 4. Metallic Implants

The modern history of metallic implants from the 18^th^ century onward is characterized by a antagonism between excellent biocompatibility of some metals (especially gold, silver and platinum) coupled with low mechanical properties and metals which exhibit good mechanical properties, especially yield strength coupled with high corrosion rates and low biocompatibility (e.g., brass, copper and iron). As a result, only a very restricted application range was possible. In the first half of the 20^th^ century, the development of modern alloys led to the advent of several materials which are still in use.

**Figure 15:**
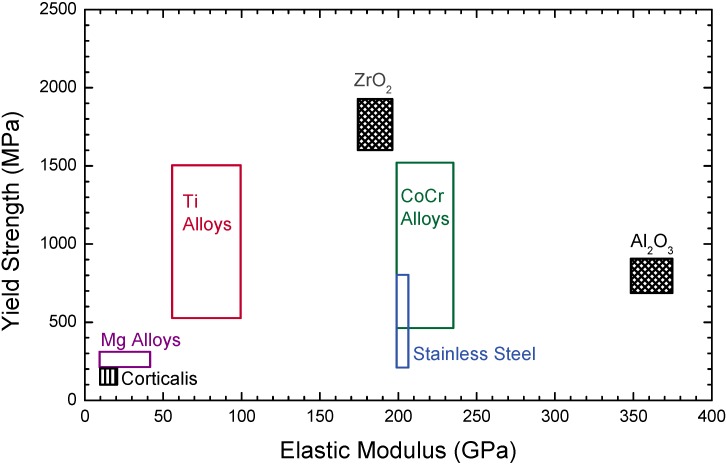
Comparison of yield strength and elastic modulus of natural bone (corticalis, calcification level of up to 90%; the softer spongiosa is calcified only for 15-25%) with metallic alloys and ceramics used for biomaterials.

The first class to be employed was austenitic stainless steel, followed by CoCrMo alloys and Ti alloys for medical uses around the middle of the last century [184]. NiTi as a shape memory and super-elastic alloy was developed in 1958 at the Naval Ordnance Laboratory. Compared to other materials, they are well known for their superior mechanical properties and biocompatibility, making them desirable as orthopedic and dental implant materials. Regarding the fatigue strength and crack growth rate in pure metals, a clear ordering of Ti → L605 → SS316L → Ti6Al4V → NiTi is observed with NiTi showing the highest rate [185]. Magnesium alloys exhibit a rather high corrosion rate, which together with high tolerance against Mg ions make them attractive for bioresorbable implants.

Beside different costs, a different combination of elastic modulus, yield strength and fracture toughness is observed in these materials (see Figure 15). An elastic modulus, which is much higher than that of bone will result in stress shielding as a stiff hip implant will change mechanical loads on the femur, thus leading to a bone atrophy [186]. Insufficient strength and fracture toughness will result in an early mechanical failure of implants. Independent of the bulk properties, all materials have been subjected to different kinds of surface modification with energetic ions [187], which will be detailed in the following subsections.

### 4.1. Titanium and Titanium Alloys

Titanium is a well established material used in artificial implants with an excellent track record as biomaterial [188]. However, in the vicinity of endoprostheses made from titanium, especially hip implants, black sludge material is often found, accompanied by aseptic loosening of the implants, which is attributed to a high wear rate and subsequent deposition of the debris near the implant [189]. Furthermore, medical implants for applications as osteosynthesis plates ‑ where more static loads or a considerably lower number of load cycles is encountered ‑ show, despite their acknowledged good biocompatibility, that an infection occurs in about 5% of the cases with a subsequent loss of the implant [190]. Thus, surface modification of Ti has a long tradition and is continuing today [191].

Using conventional coating technologies, it is possible to influence mainly the tribology of the surface, however no significant improvement of the biocompatibility was reported in some cases [192]. Tests of the biocompatibility of TiN produced by either CVD or PVD methods resulted in a classification as suited for orthopedic implants or blood-contacting material [193,194]. In addition, ion implantation with nitrogen to form TiN directly below the surface was first employed for improving the wear behavior and the surface hardness more than 20 years ago [195]. However, high costs and the complex geometries of medical implants prevented a large-scale use. In 1991, nitrogen PIII was used to treat femoral knee components made from Ti6Al4V and prove the dose uniformity for these geometries [196].

Except nitrogen and oxygen, other ion species have been investigated, too. Implantation of Nb with fluences from 0.5 to 4.0 × 10^17^ cm^-2^ at 60 keV into a Ti-Al-Zr alloy showed an increased corrosion resistance, decreasing again at higher fluences due to precipitate formation [197]. Furthermore, P [198] Ca [199,200], and Na [201] ion implantation has been shown to improve the osseointegration. The corresponding bioactive pathways are supposed to include leaching of implanted ions into the surrounding bioliquid, thus inducing the precipitation of hydroxyapatite, calcium phosphate or other compounds related with inorganic bone minerals [202].

**Figure 16:**
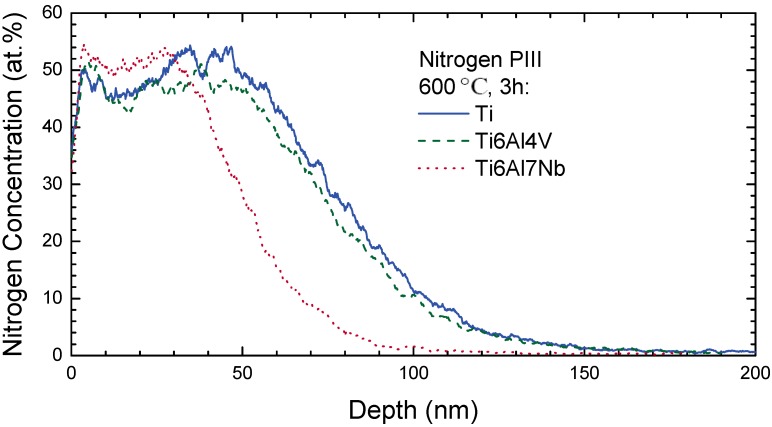
Nitrogen depth profiles after PIII into different Ti alloys; the implantation range without diffusion is about 20 nm with a straggling of 15 nm [205].

Ion implantation at low temperatures can be used to modify the near surface region of less than 100 nm at moderate fluences and energies, while diffusion assisted processes are necessary to obtain a thickness of the modified surface region beyond 1 µm. Nitrogen diffusion in Ti and Ti alloys occurs along interstitial sites within the cation matrix, leading to a phase formation tightly correlated with the texture of the base material and the formation of a compound layer [203,204,205]. However, appreciable diffusion starts only at 600 °C, depending on the alloy composition (Figure 16). The corresponding phase formation begins with ε-Ti_2_N at lower temperatures while δ-TiN dominates at higher temperatures [206,207], despite a nitrogen surface concentration of 30–35 at.% in all cases. Different activation energies and kinetics for the processes (1) Ti → ε-Ti_2_N, (2) Ti → δ-TiN and (3) ε-Ti_2_N → δ-TiN were proposed, with the activation energy increasing from (1) to (2) and (3) [203]. Using a hollow cathode discharge operating with bias voltages of 600–1,100 V, a similar phase formation regime was observed for pure Ti with the surface roughness Ra increasing from 0.2 µm up to 0.7 µm at 500 °C [208]. At the same time, the contact angle in wetting experiments decreased from 50° to values between 14° and 32° with a complex influence of process pressure and treatment time. However, aging effects were not studied.

A similar behavior is observed for oxygen implantation into pure Ti and Ti6Al4V [209,100]. For implantations at low temperatures (265–400 °C), sharp box-like edges of the oxygen profiles were found, coinciding with the calculated implantation ranges. This indicates high fluences beyond sputter saturation without any additional diffusion of the implanted atoms. With increasing temperature beyond 400 °C, two different diffusion processes can be distinguished. Oxygen ion implantation in Ti and Ti6Al4V leads to titanium diffusing towards the surface and forming a closed titania layer while oxygen diffusing into the bulk results in interstitial oxygen within the titanium matrix. Simultaneously, toxic vanadium is enriched in the surface oxide for Ti-V alloys [210]. The corresponding surface topographies have already be shown in Figure 11.

Despite the consistent diffusion mechanism across the whole temperature range, a strong influence on the phase formation, in addition to the microstructure was observed in some systems. Oxygen ion implantation into pure Ti effects always in the formation of rutile, independent of the process temperature and the heating regime [99]. For Ti6Al4V, a mixture of anatase and rutile was found, together with the formation of alumina at temperatures beyond 600 °C. Anatase, the low temperature titanium dioxide phase, is dominating when the samples are preheated to 400 °C before starting the ion implantation [100]. For medical application, the metastable high temperature Rutile phase is preferred as it exhibits a better biocompatibility than anatase, the low temperature phase [211].

The implantation process does not only influence the diffusion and phase formation processes. Different mechanical and tribological properties are expected as a function of the treatment time, temperature and heating regime as they are mediated by the layer thickness, phase composition and microstructure. Detailed investigations are available for oxygen implantation into Ti and Ti6Al4V [101,212]. Using a rotating ball-on-disc test with an alumina ball, no significant difference in the wear rate of untreated Ti and Ti6Al4V was found, whereas after oxygen implantation, a wear reduction of two orders of magnitude was found for the former material, increasing to 2.5 orders of magnitude for the latter one. In contrast, nitrogen ion implantation leads to smaller reductions of the specific wear at low load, while values similar to those of the oxides were found at the highest contact pressure of 1.4 GPa. However, as no temperature effect was found at all for Ti, the microstructure must have an additional influence, especially as thinner layers on Ti6Al4V have a lower wear rate than thicker layers on pure Ti.

In another investigation, pin-on-disc wear tests were performed against a ceramic ball for Ti6Al4V nitrided between 400 and 850 °C [213]. With increasing implantation temperature, a decrease of the friction coefficient by up to 50% was observed, while the wear was reduced by up to a factor of 10, highly correlated with the layer thickness and the occurrence of Ti_2_N. Using UHMWPE as counterbody, similar results were obtained after nitrogen PIII into Ti6Al4V with a loading bearing capability of the surface increase up to a factor of 10, however the friction coefficient remained nearly constant [214]. Implantations at high temperature with longer process times, where the intermediate compound Ti_2_N is formed before transformation into TiN, show improved fatigue properties compared to the direct formation of TiN [215].

Contrasting this implanted surfaces with TiN films deposited on Ti–6Al–4V alloy by PVD and plasma nitriding processes [216], it was observed that the PVD films, despite having higher hardness and lower surface roughness, do not provide an as good sliding wear in a dry reciprocating geometry against a ruby ball as the plasma nitrided samples. In the latter samples, beside a predominant Ti_2_N phase, a diffusion layer below the nitrided layer allowing a better mechanical support for the surface region was observed, which should account for the difference in the results.

Using a more realistic wear test in hip simulators, a reduction of the wear volume by one third was found in modular hip implants [217], whereas a reduction of 75% was observed for implants tested against bone cement [218]. Here, a strong influence of the layer thickness is found as the wear rates apparently increase with the trial during. Actually when the modified layer has been removed, the high wear rate of the base material is established. Additionally, a very strong effect of the composition of the bone cement exists with the presence of hard zirconia particles aggravating the wear behavior for Ti- and Co-based THRs. Nevertheless, even TiN PVD coated Ti and Ti alloys [219,220] showed reduced fretting fatigue damage in hip implants and bone plates.

**Figure 17:**
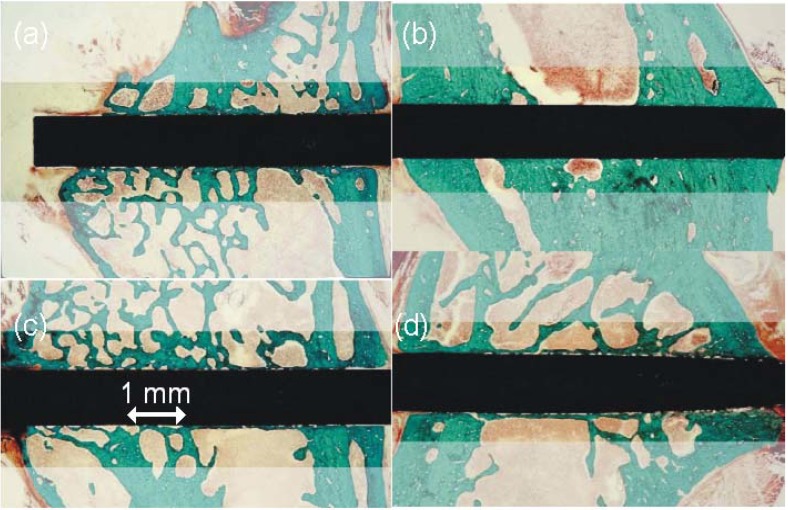
Histological cross sections [223]: (**a**) pure Ti, untreated: few gaps. (**b**) pure Ti, after PIII: complete osseointegration. (**c**) NiTi untreated: large gaps. (**d**) NiTi after PIII: good osseointegration.

Animal tests of oxygen PIII treated implants were conducted using a rat model, introduced by the university of Würzburg [221]. The osseointegration was investigated by biomechanical pull-out tests as well as histological investigations and fluorescence microscopy [222]. Compared to THRs, a reduced total wear is observed in these environments as the loading conditions are less severe. After oxygen PIII treatment, a much improved osseointegration (see Figure 17) was found for titanium, together with considerably higher pull-out forces [223]. Similarly, the surrounding tissue reaction to NiTi implants, as measured with fluorescence markers, is reduced by about 50% [224]. As a primary effect, a better osseointegration correlated with the nanotopography can be envisaged. However, additional effects from an enhanced apatite formation on the modified surface cannot be ruled out.

Explanted TiN-coated hip prosthesis heads showed the presence of a phase of calcium phosphate on more than 50% of the explants [225]. It was noted that this behavior, not observed for naturally passivated titanium surfaces, can be ascribed to the presence of an oxynitride phase, TiO_x_N_y_, formed *in-vivo* during the severe wear conditions encountered by the hip prosthesis heads. Similarly, sodium implantation into titanium [201] shows a difference between beamline implantation and PIII with the bioactivity, as determined by precipitation of calcium phosphate and cell proliferation with the PIII samples more bioactive than the beamline implanted samples, where less sodium is residing in the surface layer. Additional investigations into the surface roughness and profile determination could help to elucidate the underlying effect.

These investigations on improving the biocompatibility of Ti and Ti6Al4V (together with other titanium alloys) centered initially on the tribology and wear resistance, with the bioactivity enhancement through the modified topography a (fortuitous) coincidence. Nowadays, the investigations concentrate mainly on the topography while exploiting the concomitant improvements of the surface mechanics. The corrosion rate itself has only been an afterthought, except for selected experiments where the leaching of selected compounds was exploited to augment the formation of hydroxyapatite on the surface. In contrast, the majority of investigations on NiTi focus on improving the corrosion properties, respecting the increased retention of toxic Ni ions inside the material.

Despite the widespread use of NiTi as a biomaterial [115], there are still no conclusive data on the biocompatibility of NiTi itself, with several studies showing that NiTi alloy is safe to use [226,227]. As an intermetallic compound, the binding energy of Ni and Ti atoms is quite high, except for grain boundaries or defects introduced by any mechanical treatment. Normal sterilization procedures using autoclaving actually improves the quality and thickness of the native surface oxide, thus reducing the Ni release rate [16]. Oxygen ion implantation at elevated temperature further improves this oxide barrier as Ni is preferentially sputtered from the surface and Ti is diffusing towards the surface to form a pure TiO_2_ surface oxide [228,229]. Using nitrogen ion implantation, a similarly reduced Ni release and improved cytotoxicity has been found [230].

### 4.2. Stainless Steel

Austenitic stainless steel is well known for its excellent corrosion resistance properties, resulting in its early use in fixed implants such as for artificial joints and temporary fixation devices. However, very high mechanical wear rates, together with selective, low-level Ni and Cr release when the surface is damaged and higher susceptibility to pitting corrosion than Ti or CoCr alloys reduced the reliance on steel for permanent implants [164,231]. While gas nitriding or plasma nitriding are common procedures for close to 100 years to improve the surface hardness of low-alloy steels [232], nitriding of austenitic stainless is complicated by a thick native oxide layer, presenting a diffusion barrier against nitrogen insertion, the occurrence of CrN at temperatures beyond 450 °C, resulting in high corrosion rates, and core softening at temperatures around 450–400 °C [233].

Using ion implantation at different ion energies in the temperature range from 350–450 °C, nitriding and carburizing of austenitic stainless steel became possible about 30 years ago. Employing this process, the formation of a very hard and wear resistant layer with an unusual high nitrogen content of up to 35 at.% in solid solution and a corresponding lattice expansion of some 5–15% is observed. No nitride layer (ε or γ’) is accompanying this structure. This phase, generally called expanded austenite [234], can be obtained by a wide range of methods in the narrow temperature range, by plasma nitriding [235,236,237] low-energy high-flux ion implantation [238,239], PIII [234,240] as well as conventional ion implantation [241]. Similar diffusion constants are published for all methods, albeit slightly lower activation energies were obtained for plasma nitriding [235,241].

**Figure 18:**
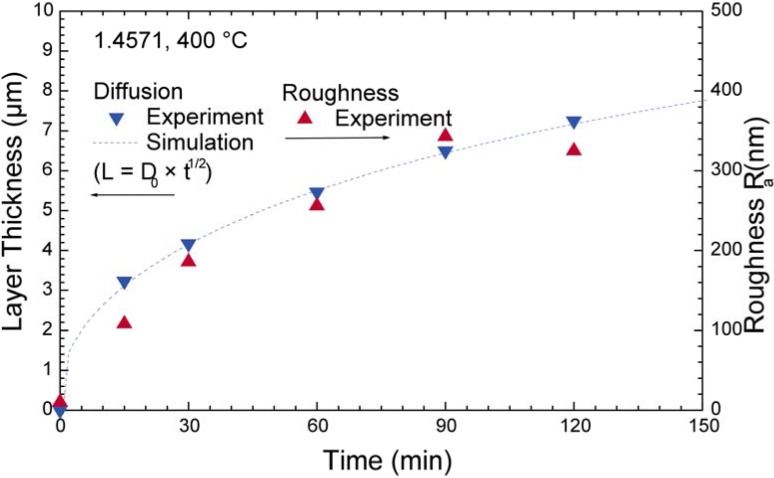
Evolution of layer thickness and surface roughness with time for PIII nitriding of austenitic stainless steel 1.4571 (X6CrNiMoTi17.12.2).

When comparing treatments for different times and at different temperatures, a thermally activated diffusion is observed with either a linear (at low nitrogen concentrations) or inverse parabolic (at higher nitrogen concentration) layer growth. Correspondingly, the surface roughness increases with increasing ion flux, as shown in Figure 18. To explain this diffusion behavior, a model assuming trapping of nitrogen at Cr sites was developed and refined [241,242]. Once all chromium trap sites are occupied, the additional incoming nitrogen can diffuse rapidly through the saturated layer. Inserting a activation energy for diffusion of 1.1 eV and for detrapping of 1.45 eV, a very good agreement with the measured depth profiles, even for subsequent ^14^N/^15^N implantations is observed [243].

However, one chromium atom is supposed to trap more than one nitrogen atoms as a chemical composition of Fe_0.46_Cr_0.12_Ni_0.07_N_0.35_ is present for high nitrogen concentrations. An alternative model assumes a step-like increase of the nitrogen diffusivity after a certain threshold in the lattice expansion is observed [244]. A maximum occupancy of the octahedral interstitial sites of 61%, corresponding to 38 at.% nitrogen was found when nitriding thin steel foils [245]. At the same time, a less tightly binding configuration was inferred from the data for N/Cr ratios higher than 1:1.

**Figure 19:**
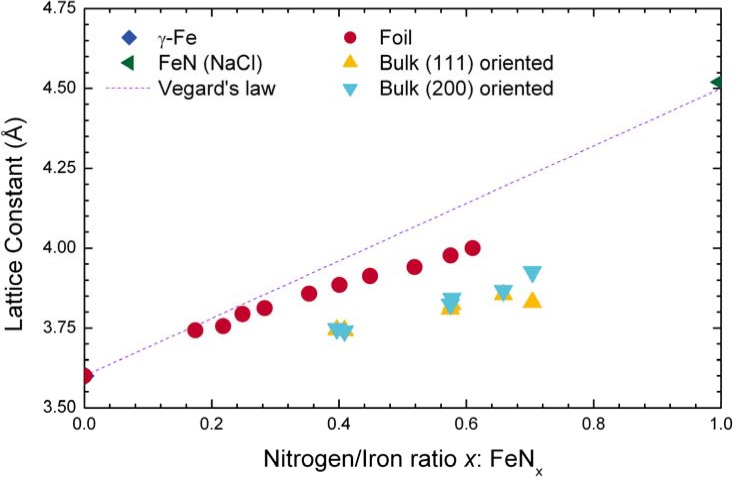
Lattice expansion vs. nitrogen content for nitrided thin foils [245] and surface nitrided bulk material [249]. Additionally, a linear relation (Vegard’s law) with NaCl-type FeN [250] as endpoint is shown for comparison.

While an isotropic lattice expansion is observed for completely nitrided thin foils, an anisotropic expansion is measured after superficial nitrogen insertion into bulk material, depending both on the orientation relative to the substrate surface and the crystallite orientation [246]. The exact crystallographic description of such layers is still not available [247]. When comparing the lattice expansion normal to the surface with data for thin foil and Vegard’s law, lower lattice constants are found for nitrided surfaces (see Figure 19), which may be explained by additional stress inside these surface layers [248].

**Figure 20:**
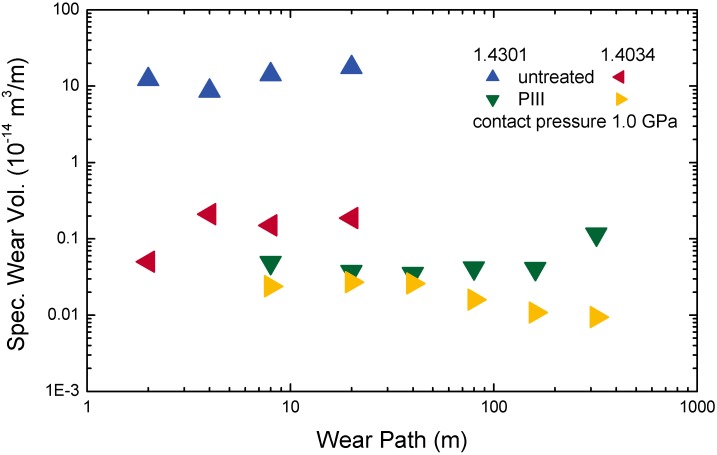
Specific wear volume as a function of the wear path for untreated and implanted (350 °C, 2 hours) 1.4034 (martensite/X46Cr13) and 1.4301 (austenite/X5-CrNi18.10) against a WC ball [251].

The hardness of the expanded austenite layers reaches values up to 12 GPa. A strongly reduced wear is correlated with this hardness increase. In contrast to untreated stainless steels, rather high loads, standing for high contact pressures of 0.75–1.0 GPa, are necessary to measure any wear at all. Figure 20 shows results obtained in oscillating ball-on-disc geometry with a WC ball at different wear paths [251]. Comparing the results, austenitic stainless steel reaches higher hardness values than and similar wear rates as standard martensitic steel.

Dissimilar to the vast volume of research on ion implantation into austenitic stainless steel, only a handful of reports on the biocompatibility of such materials is freely available. Sputter coated steel 316L does exhibit a strongly reduced biocompatibility [252], whereas plasma nitriding or ion implantation results in a similar or an improved biocompability [253,254]. Oxygen ion implantation into steel 316LVM showed that bare metals stents can be as efficient as more expensive drug eluding stents in the treatment of low- to medium-risk lesions with low incidence of angiographic restenosis [255]. In all these cases, the mechanical and tribological surface properties were improved, leading to a reduced wear rate, whereas the corrosion rate is affected by the ion species. For oxygen, an improvement can be obtained, whereas the results for nitriding, respective nitrogen implantation depend critically on the process temperature and time. For longer processes and higher temperatures, detrimental CrN formation is obtained [256]. Effects of the surface topography have not been explicitly investigated for the osseointegration of stainless steel after ion implantation.

### 4.3. CoCr Alloys

The first generation of metal-on-metal THRs was supplanted by polyethylene bearings by the mid 70s as the pure metal implants showed high clinical failure rates. Due to improved quality, a second generation with relatively low volumetric wear, compared to metal-polyethylene and ceramic polyethylene, is increasingly accepted. The biocompatibility of CoCrMo alloy is related closely to this material's excellent corrosion resistance, imparted by a thin passive oxide film that forms spontaneously on the alloy surface. X-ray photoelectron spectroscopy (XPS) analysis reveals that its composition is predominantly Cr_2_O_3_ oxide with some minor contributions from Co and Mo oxides [257]. Nevertheless, particle generation and subsequent release of toxic Co ions from them, facilitated by very large surface/volume ratios are still considered problematic [116,258].

Conventional coating techniques have been employed to form hydroxyapatite with an increased bioactivity [259], while the adhesion of diamond-like carbon (DLC) films was increased from 2.8 to 39 MPa using plasma based ion implantation and deposition [260]. Results after ion implantation, either by conventional broad beam implantation or PIII, are sparsely reported compared to austenitic stainless

After observing a significant reduction in polyethylene wear for nitrogen ion beam implantation into Co-Cr femoral heads [261], a follow-up study was conducted by another group using PIII for nitrogen implantation into a cobalt-chromium-phosphorous alloy [262]. Essentially two different sets of process parameters were investigated: respective 10 and 20 kV for 1 and 2 hours at two temperatures which should have been near 100 and 250 °C, as estimated from the reported plasma data. The surface roughness *R_a_* remained approximately constant at 40–50 nm during the implantation. For the higher temperature, a reduction of the friction coefficient against ultra-high molecular weight polyethylene (UHMWPE) from 0.15 to 0.10 was observed with early failure of the low temperature samples.

High intensity plasma ion nitriding was used to modify cast and forged CoCrMo between 300 and 800 °C for 3 hours with acceleration voltages between 0.4 and 1.5 keV to investigate the tribology [213] and microstructure [206]. The surface roughness *R_a_* increased with increasing process temperature to values of 30–300 nm. Friction against a ceramic ball showed no significant effect of the nitrogen implantation, whereas a reduced wear was observed only for the forged CoCrMo alloy. A complex phase formation pattern leading to CrN, Cr_2_N and the σ-phase, depending on the treatment temperature and the ratio of the hexagonal ε-phase to the fcc γ-phase was detected. The ε/γ ratio is different for the cast and forged alloy due to their dissimilar chemical composition. Precipitates may occur at selected temperatures, otherwise the microstructure and grain size of the parent phase are maintained while a modified surface layer of 3–5 µm is formed.

**Figure 21:**
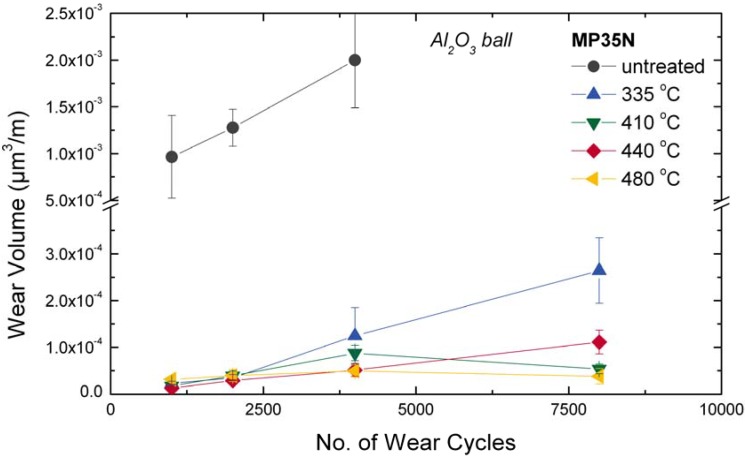
Wear volume as a function of number of wear cycles at room temperatures and preceding PIII at different temperatures for CoCr alloy MP35N. Please note the axis break [263].

Using PIII to insert nitrogen ions into different CoCr alloys, at moderate temperatures between 300 and 500 °C, the formation of an expanded austenite lattice, similar to austenitic stainless steel was observed, together with anomalous profile shapes and fast diffusion [264,265,266]. The surface roughness *Ra* increased from about 10 nm for the polished specimen up to 100 nm. At higher temperatures, precipitation of CrN and Cr_2_N was observed. Concurrent with a nitrogen content of up to 25 at.%, situated on interstitial sites, a hardness increase up to 20 GPa and a wear reduction up to a factor of four were observed (see Figure 21), nearly independent of the process temperature and surface roughness [263].

Similarly, broad beam nitrogen ion implantation at 60 kV at temperatures from 100 to 400 °C for 30 minutes leads to the formation of an expanded phase for medical grade Co26Cr6Mo [267]. Using a static immersion test in SBF, an initial burst of Co release was observed, reaching the low value for untreated control samples after about a week. Apparently, the insertion of nitrogen leads to some preferential bonding structure between Cr-N, thus weakening the original CoCrMo alloy. Hence, hard and wear resistant surface layers can be formed, but apparently only at the price of increased leaching of weakly bound Co atoms. A careful balancing between wear rate and corrosion resistance has to be performed to find a compromise depending on the actual application. Alternatively, oxygen implantation resulting in very thin modified surface layers with a wear rate reduced by 50% and some progress on corrosion inhibition [268]. Furthermore, detailed investigations of additional influences of the topography are necessary.

### 4.4. Mg Alloys

In contrast to the previous subsections, the release of magnesium ions as a corrosion product can be viewed as physiologically beneficial. At an average content of about 30 g Mg for an adult human [269], a recommended dietary allowance of 320, respective 420 mg/day is given by the US Food and Nutrition board for males and females [270]. Furthermore, Mg is facilitating the mineralization of bone tissue through its strong binding to phosphates. At the same time, an elastic modulus of pure Mg close to that of bone, albeit with a higher yield strength is observed (see Figure 15), thus early uses in trauma surgery date back to 1907 [271,272]. Nevertheless, excessively large corrosion rates of 0.5–50 mm/year are still restricting the widespread use of unprotected Mg alloys—for biomedical as well as other applications as structural material [36,273]. A fast corrosive dissolution at the physiologically present pH of 7.4–7.6 and the high chlorine concentration is generally accompanied by hydrogen evolution, however it is reasonable to believe that this does not constitute a major problem [274,275].

**Figure 22:**
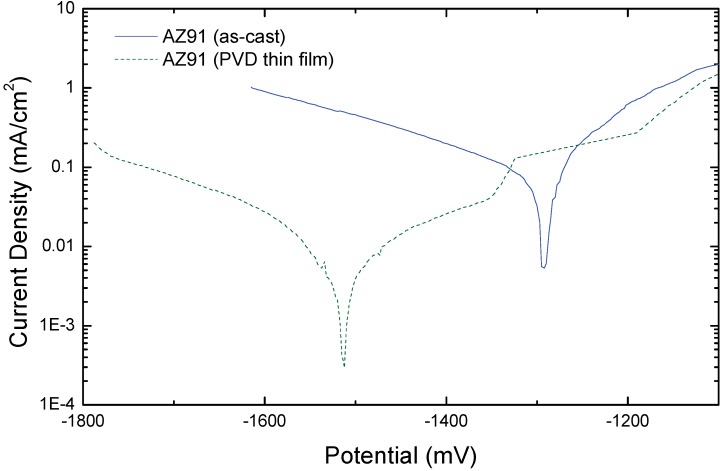
Current versus potential curves of as-cast and AZ91 alloy deposited by ion beam sputtering (PVD thin film). The EIS measurements were performed in 0.5% NaCl solution at pH 11 [276].

Conventional alloy development to reduce the corrosion rate focuses on three approaches. First, detrimental elements such as Fe, Ni and Cu have to be reduced as far as possible as they accelerate micro-galvanic corrosion [277]. Second, alloying elements, which retard the biodegradation and are biotolerable or show at best only a small toxicity, such as Zn, Mn, Ca and rare earth elements can be employed [278,279,280]. Thirdly, a fine microstructure or an amorphous structure are also beneficial for lower corrosion rates [277,281,282].

As a surface modification, several different types of coatings have been employed to reduce the corrosion rate [36]. In addition, ion plating [283] or ion implantation [284] with Ti reduces the corrosion current density. Alternatively, coating with Mg or Mg alloys using PVD techniques treatments can prolong the dissolution phase [44]. Besides a lower corrosion rate, these novel thin films are characterized by a rather homogeneous dissolution, thus avoiding the enrichment of more noble alloying elements and residual precipitates being converted in non-soluble particles. Both of these effects are highly detrimental for the use of Mg-alloys as resorbable biomaterials. In principle, this concept can be transferred to any available Mg alloy system.

**Figure 23:**
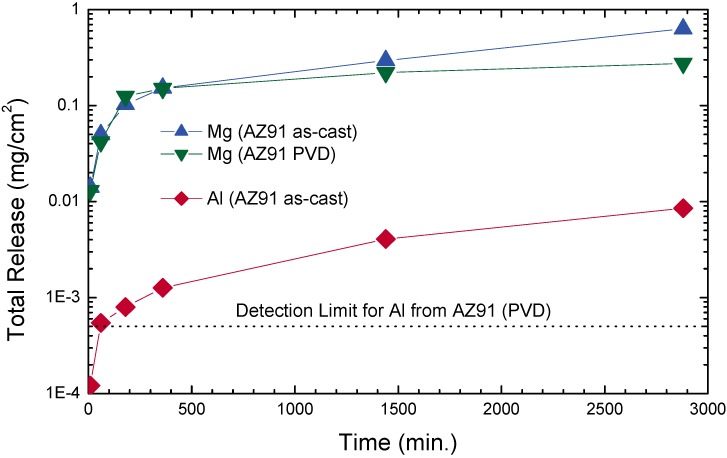
Integrated release rates for Mg and Al from AZ91 alloy in as-cast condition and as PVD thin film (immersed in 1% NaCl solution). The release rate for Al from the thin film is below the detection limit.

An example for such microstructures has already been shown in Figure 3. These highly textured films exhibit a shift of the free corrosion potential by about –200 to –300 mV (see Figure 22), thus offering the possibility of cathodic protection, especially for pure Mg and Mg-Al alloys [285]. A much lower and more homogeneous dissolution was found for the thin film coatings [286], compared to the as-cast alloys (see Figure 23), which consist exclusively of the α-phase with all alloying elements or impurities in solid solution. The formation of passive films is much easier on these coatings, visible in a reduced weight loss rate at longer immersion times. The incorporation of Al and/or Zn obviously stabilises the passive film based on Mg(OH)_2_ thus offering higher corrosion protection for the AZ91D coating compared to the pure Mg coating. The pure Mg coating has a similar corrosion resistance as the bulk AZ91D alloy [286]. Thus, a superior biocompatibility can be postulated for these PVD coatings as the most critical effects of as-cast alloys are avoided: A less pronounced release of potentially toxic Al and rare earth ions as well as the release of metallic particles which will be distributed throughout the whole body and be subjected to increased corrosion rates when liberated from the Mg matrix.

## 5. Summary and Conclusions

It is possible to modify and improve the biocompatibility of metals using energetic surface treatments. However, the correlation between ion implantation parameters and biocompatibility is rather indirect and complex with many different intermediate levels. Nowadays, most of these intermediate steps, especially in the biochemical interaction of metallic implants with living tissue are rudimentary understood, while further investigations are still necessary. Nevertheless, synergistic effects have been shown, with corrosion, release of wear particles and surface topography modified simultaneously. These results encompass all major classes of metallic alloys employed as biomaterials. For titanium alloys, the most knowledge is available, together with more possibilities to modify the surface topography as outward diffusion and phase formation processes leading to oxides and nitrides are active. In contrast, stainless steel and CoCr alloys require finely tuned compromises as an increased surface corrosion and release of toxic elements is quite easily obtained during certain experimental conditions. Biodegradable magnesium alloys have become a hot topic, however no clearly favored alloy composition or surface modification is presently perceptible.

However, the list of possible applications is far from complete, with further additions certain to be seen in the future. For instance, the artificial differentiation between different groups of applications, e.g. cardiovascular and orthopedic, could be removed. By integrating selective signaling towards several cell types into the surface, a better biointegration will be achievable as angiogenesis and osseointegration are both necessary in orthopedic application, together with an interaction with the immune system. In other applications, the same cell types, albeit at different ratios are be present.

Regarding the translation from laboratory results towards clinical applications, restricted geometries or the requirement of masks to create surface structures are not limiting the application of energetic surface bombardment or hindering the transfer towards large scale manufacturing. Why do we presently see only few investigations in this interesting field of interdisciplinary science? First, an interdisciplinary collaboration between materials scientists and biochemists or clinical researchers is fundamental for such research work. Furthermore, a rather comprehensive surface characterization describing chemistry, tribology and topography necessitates either a extremely widespread analytical department or collaborations between different groups is mandatory to be able to benefit from the underlying synergy effects where several surface features are addressed within one experiment. Similar grouping of experience is also necessary on the biological side. Thus, even limited investigations require a large effort in people and resources.

Second, ion implantation is still a process closely attached to semiconductor industry with equipment dedicated to metallurgical applications as a niche market. Equipment for PVD or CVD coatings, in contrast, is already past commoditization supplying a mass market across different industries. However, the surface topography obtained there is always tending towards smoother surfaces, covering the underlying topography of the substrate. For high rate processes with low surface mobilities, highly structured or porous surfaces are possible, e.g. for hydroapatite coatings formed by plasma spraying [287]. In all these applications, deposition or coating processes dominate over sputtering, thus no real structuring of the substrate itself occurs, in addition to the possibility of adhesion problems or residual stresses. Additional substrate biasing or the inclusion of external ion sources to the existing coating equipment, which would change the focus from deposition towards structuring are rather complex and time consuming.

At the same time, the available aspect ratios by physical or physical-chemical sputtering using ions or a combination of ions and reactive gases are limited, when compared to alternative, purely chemical etching processes [34,35]. By purely chemical processes without vacuum or ion bombardment, related surface topographies [288] as well as high aspect ratio topographies [289] or even 3D-scaffolds can be produced [290]. Using such chemically prestructured substrates for subsequent physical surface functionalization by energetic ions could lead to highly promising results. Additional combinations of ion bombardment with subsequent biografting are also possible, albeit beyond the scope of this review.
